# Whip spiders (Amblypygi) become water-repellent by a colloidal secretion that self-assembles into hierarchical microstructures

**DOI:** 10.1186/s40851-016-0059-y

**Published:** 2016-11-28

**Authors:** Jonas O. Wolff, Thomas Schwaha, Michael Seiter, Stanislav N. Gorb

**Affiliations:** 1 Functional Morphology and Biomechanics, Zoological Institute, University of Kiel, Am Botanischen Garten 9, Kiel, 24098 Germany; 2 Department of Biological Sciences, Macquarie University, Sydney, NSW 2109 Australia; 3 Department of Integrative Zoology, University of Vienna, UZA1 Althanstraße 14, Vienna, 1090 Austria; 4 Institute of Zoology, Department of Integrative Biology and Biodiversity Research, University of Natural Resources and Life Sciences, Gregor Mendel Straße 33, Vienna, 1180 Austria

**Keywords:** Anti-wetting, Surface coating, Cuticle, Colloid, Arachnida, Amblypygi, Plastron, Cerotegument

## Abstract

**Background:**

Among both plants and arthropods, super-hydrophobic surfaces have evolved that enable self-cleaning, locomotion on water surfaces, or plastron respiration. Super-hydrophobicity is achieved by a combination of non-polar substances and complex micro- and nano-structures, usually acquired by growing processes or the deposition of powder-like materials.

**Results:**

Here we report on a multi-phasic secretion in whip spiders (Arachnida, Amblypygi), which externally forms durable, hierarchical microstructures on the basically smooth cuticle. The solidified secretion crust makes the previously highly wettable cuticle super-hydrophobic. We describe the ultrastructure of secretory cells, and the maturation and secretion of the different products involved.

**Conclusion:**

Whip spiders represent intriguing objects of study for revealing the mechanisms of the formation of complex microstructures in non-living systems. Understanding the physical and chemical processes involved may, further, be of interest for bio-inspired design of functional surface coatings.

**Electronic supplementary material:**

The online version of this article (doi:10.1186/s40851-016-0059-y) contains supplementary material, which is available to authorized users.

## Background

Water-repellence is an important property for various biological surfaces, avoiding unwanted wetting, contamination, water-loss, fouling and conglutination, and permitting self-cleaning effects, locomotion on water surfaces, and plastron respiration [1–6]. While hydrophobicity by surface chemistry can achieve a maximal water contact angle (CA) of ~120°, the super-hydrophobic state (CA > 150°) is achieved by additional microstructuring of the surface [7]. Because small water droplets form a nearly spherical shape, they roll off the surface at a tilted angle below 10° [8]. The report of this phenomenon in plants in the late 1990s [1] generated enormous interest in water-repellent and self-cleaning surfaces, and led to innovations in artificial super-hydrophobic materials and surface coatings [9, 10]. However, there are many drawbacks in the production of such materials, and especially the durability of such coatings is often not satisfying [10].

In nature, the necessary micro- and nano-roughness of the surface is produced in very different ways (Table 1). Micro- and nanostructures can be grown, like dense pads of hydrophobic hairs (setae and/or microtrichia) in water-walking insects and spiders [3–6], granulated epicuticular structures in springtails [11–13] or the epidermal microstructure of plants [2]. They may be produced by rod- or platelet-like wax crystals that are extruded through the cuticle, as in insects and plants [1, 14–17], or arise from a secretion that is actively applied and contains intracellularly formed microparticles, as in leafhoppers [18–20].

**Table 1:** Overview of different mechanisms that produce superhydrophobic surfaces (Water-CA > 150°) in plants and animals

Organism	Principle	References
Plants		
lotus (*Nelumbo nucifera*)	wax crystals on nubby epidermis	[1]
nasturtium (*Tropaeolum majus*)	tubular wax crystals	[2]
Insects		
damselfly wing (*Calopteryx splendens*)	rod-like wax crystals	[30]
sawfly larva (*Rhadinoceraea micans*)	wax crystals on nubby cuticle	[17]
butterfly wing (*Papilio xuthus*)	microstructured scale-like setae	[31]
water strider (*Gerris remigis*)	grooved setae	[3]
backswimmer (*Notonecta glauca*)	setae and microtrichia	[6]
springtail (*Tetrodontophora bielanensis*)	granulated epicuticle	[12, 13]
leaf hopper (*Athysanus argentarius*)	nanoparticle impregnation	[18]
Arachnids		
fishing spider (*Dolomedes triton*)	setae with lipid coating	[4]
whip spider (Amblypygi)	granulated secretion coat	this study

Whip spiders (Arachnida: Amblypygi) are tropical or subtropical arachnids living in damp places, such as caves, leaf litter or under tree bark [21]. Weygoldt, wjp was the first and the only one who comprehensively study the biology of these cryptic arachnids, noted a ‘clay-like’ powder on the cuticle of whip spiders [21]. However, further data on this ‘powder’ are lacking. It has been reported that whip spiders are able to survive submerged due to plastron respiration [22], however the physical mechanism of plastron formation remained obscure. It was proposed that the wrinkled structure of the cuticle close to the book lungs is responsible for water-repellence and air-entrapment [22]. However, plastron-forming structures are usually more complex and show roughness on finer length scales [6]. Gland openings “of unknown function” have been repeatedly found on the cuticle of whip spiders [21, 22]. This may indicate the importance of secretions for waterproofing. We hypothesize that the granular coating reported by Weygoldt [21] is a secretion product that is responsible for the entrapment of air and a repellence of water. In the present study we sought to test this hypothesis and to reveal the fine structure and origin of this substance.

## Methods

### Study animals

We investigated the ability to repel water and the surface structure of the carapace in the following species of whip spiders: *Charinus acosta* (Quintero 1983) from Artemisa-Cuba (Charinidae), *Charon* cf. *grayi* (Gervais 1842) from Negros-Philippines (Charontidae), *Damon annulatipes* (Wood 1869) from Durban-South Africa, and *Phrynichus ceylonicus* (C. L. Koch 1843) from Beliluhoya-Sri Lanka (Phrynichidae); *Paraphrynus carolynae* Armas 2012 from Arizona-USA, *Phrynus longipes* (Pocock 1894) from Peninsula Samaná-Dominican Republic, and *Phrynus decoratus* Teruel & Armas 2005 from Cienfuegos-Cuba (Phrynidae). This covers all extant whip spider families, except Paracharontidae, which are nearly unobtainable [21]. Study animals were wild caught or bred from wild-caught animals, and kept in plastic- and glass terraria using standard methods [21]. Temperature was kept constant at 26–27°C and relative humidity varied between 65 and 75%. Animals were fed every seven days with cricket nymphs (*Acheta domestica*) in suitable sizes.

### Wettability tests

To test the wettability of the whip spider cuticle, 30 μl droplets of tap water were dripped on the carapace (dorsal prosomal shield) from a height of 1–5 cm (depending on the size of the animal) using a micro-pipette. Tap water had the following characteristics: conductance 448 μS; GH 14°; KH 10,5; pH 8,0 NH_4_ < 0,05 mg/l; NO_2_ 0,025-0,05 mg/l; NO_3_ 1,0 mg/l; Fe 0,02-0,05 mg/l; PO_4_ < 0,02 mg/l; SiO_2_ 2,0-3,0 mg/l; Mg 10,0; K 2,0 mg/l. In a second test series, two days later, droplets were carefully placed on the carapace, and we observed whether they remained attached or rolled off when the animal moved. Both nymphs and adults were tested, with 3–15 individuals per species.

### Microscopy

For microscopy studies, pieces of freshly collected, air dried exuviae (carapace and femur leg IV) were used. To study the secretion process we studied two animals of *C. acosta*, with one killed (~2 min in a deep-freezer at –20°C) ~2 h after moulting, and another one ~30 h after moulting. The specimens were cut in half, and one piece was air dried and the other one was fixed in 2% glutaraldehyde solution in 0.1 M sodium-cacodylate buffer. Afterwards specimens were rinsed in buffer and postfixed in 1% osmium tetroxide for approximately one hour before rinsing 3–4 times in double-distilled water. Samples were afterwards dehydrated via acidified dimethoxy-propane (DMP) for 30 min followed by three washes in acetone. Furthermore, a freshly ablated, air dried leg of a *P. longipes* 6 h after moulting, and pieces of carapace and walking legs of the same individual deep frozen 24 h after moulting, were studied. For light microscopy images a multifocal stereo microscope (Leica M205 A, Leica Microsystems GmbH, Wetzlar, Germany) equipped with a camera (Leica DFC420) was used. For scanning electron microscopy, samples were sputter coated with 10 nm Au-Pd and viewed in a Hitachi S-4800 SEM (Hitachi Ltd., Tokyo, Japan) at an acceleration voltage of 3.0 kV. Samples of moulted *C. acosta* were sputter coated with 20–40 nm gold and examined in a JEOL IT300 SEM (JEOL, Akishima, Japan) at 20 kV.

#### Histological and cytological processing

Freshly moulted and inter-moult specimens of *C. acosta* were treated as described above, including the complete dehydration step in acetone before being embedded into Agar Low Viscosity Resin (LVR, Agar Scientific, Stanstead, Essex, UK). Cured resin blocks were sectioned at 1 μm (semithin) or 60 nm (ultrathin) on a Leica UC6 ultramicrotome (Leica Microsystems, Wetzlar, Germany). Semithin sections were stained with toluidine blue for few seconds on a heating plate at 60 °C, whereas ultrathin sections were contrasted with uranylacetate for 30 min and leadcitrate for 5–10 min. Light microscopy sections were viewed with an Olympus BX53 equipped with a DP73 microscope camera (Olympus, Tokyo, Japan). Ultrathin sections were analyzed with a Zeiss Libra120 TEM (Zeiss, Oberkochen, Germany).

## Results

### Structure of the cuticle coating

**Fig. 1:**
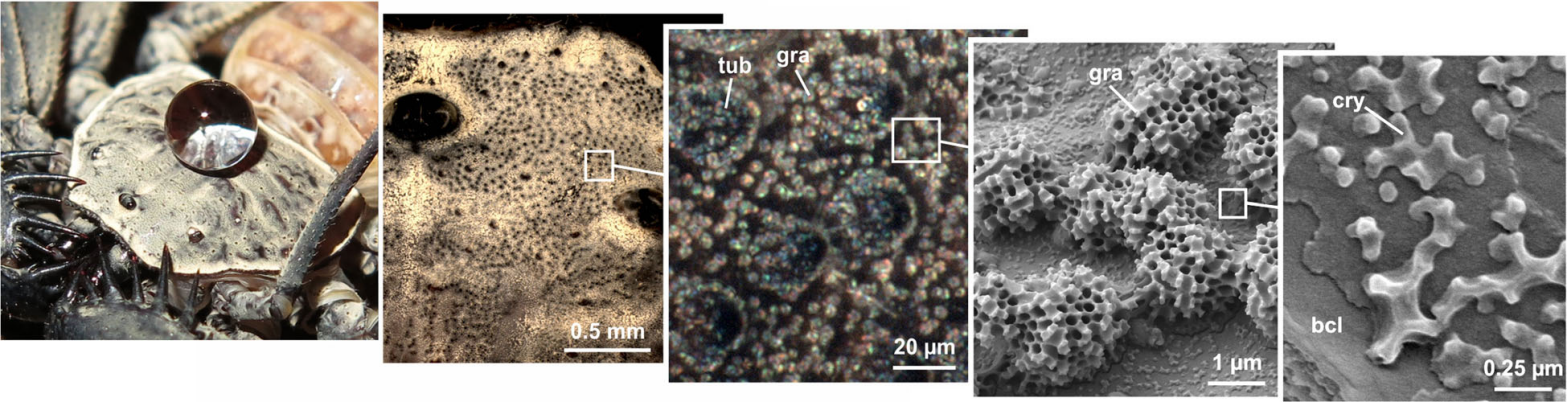
Effect and structure of the cuticular secretion in *Phrynus decoratus*. Water dripped from above onto the carapace forms a perfect droplet and rolls completely off (see also video Additional file 2: Video S1). Close inspection of the cuticle reveals a white ‘powder’ assembled on and between tubercles (tub), consisting of globular particles, called granules (gra) (from *left* to *right*). The granules arise from a colloidal secretion and may contain crystalline particles (cry). The underlying cuticle (bcl) is smooth

**Fig. 2:**
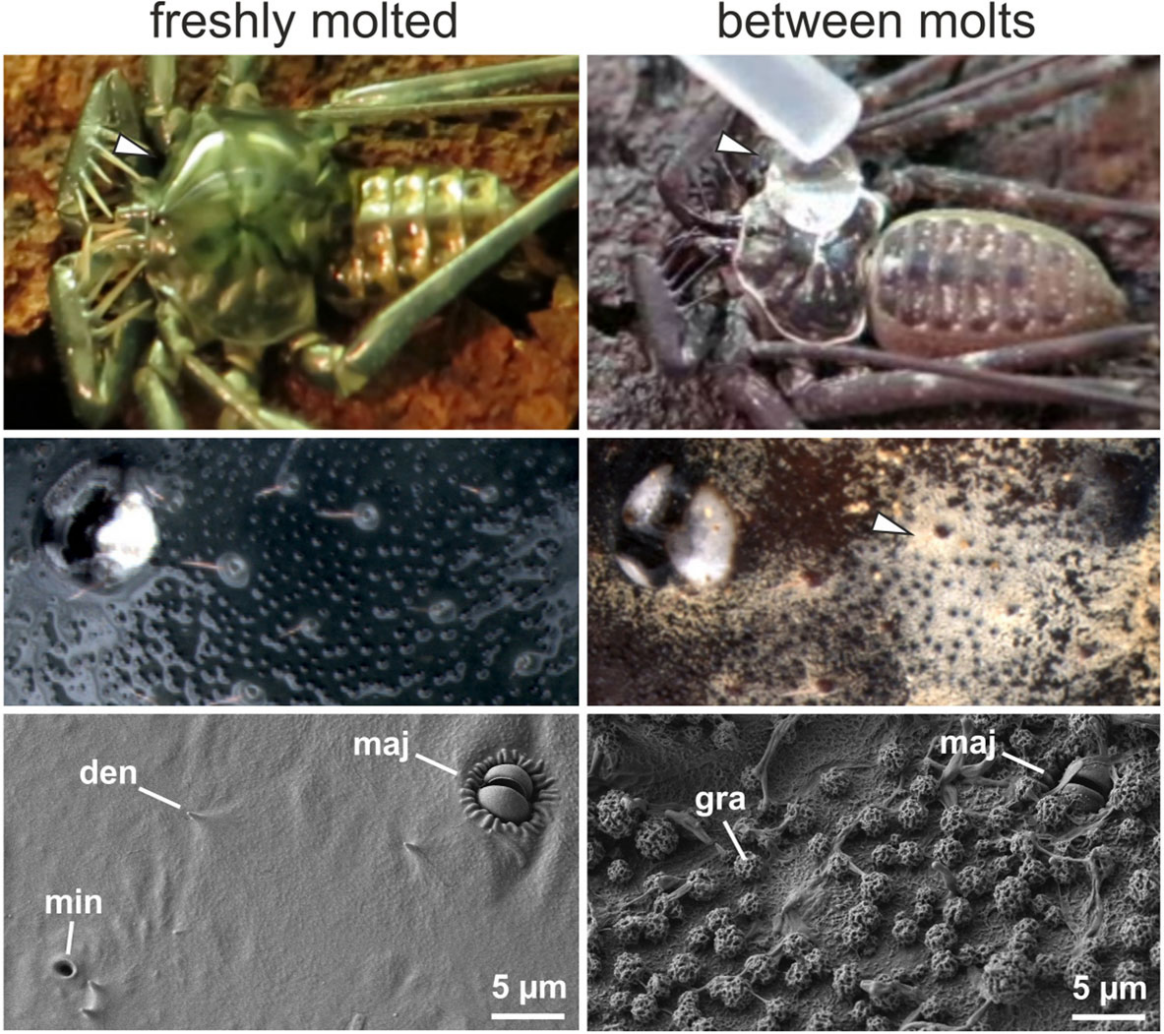
Difference between the cuticle before and shortly after moulting in *Phrynus longipes*. Some hours after moulting (*left column*) the cuticle is highly wettable by water droplets (*arrowhead*) (see also video Additional file 3: Video S2). The cuticle lacks the granules and is rather smooth on the microscale, except for small denticles (den) that help in arresting the later secretion layer. There are two different gland openings, the 2-lipped major (maj) and the pore-like minor (min) one. Between moultings (*right column*), water droplets are completely repelled (*arrowhead*) by the cuticle. The secretion layer is visible, as white ‘powder’ (*arrowhead*), composed of wrinkled granules (gra), creating a micro- and nano-roughness

**Fig. 3:**
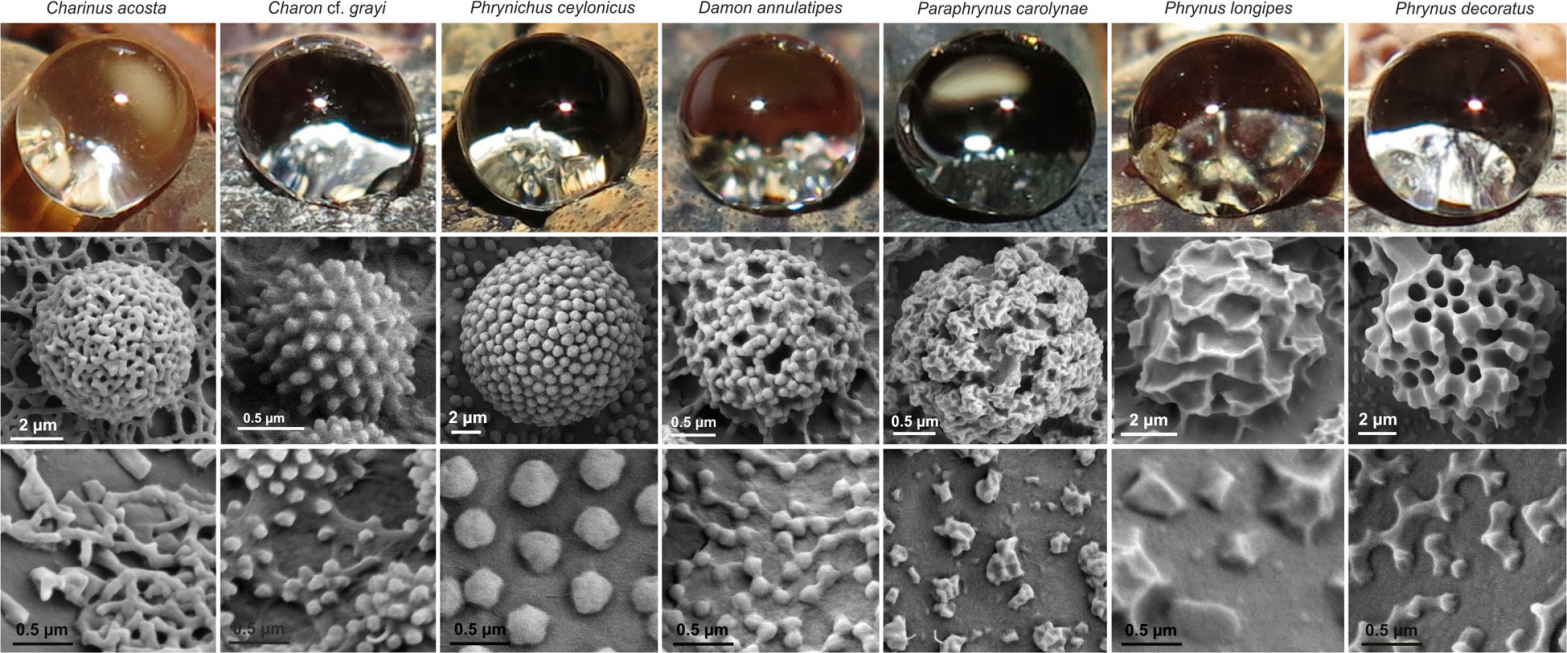
Water repellence and ultrastructure of granules in different species of whip spiders. 1^st^ row: Tap water droplets, directly ejected on the carapace, form a spherical shape in most species. 2^nd^ row: The fine structure of granules is highly species-specific. 3^rd^ row: The ultrastructure of colloid particles differ between species

We found that the whip spider integument is covered with a crust of a solidified secretion that forms globular microstructures (granules) with a diameter of 0.3–5.0 μm. Granules may exhibit a coat of assembled crystal-like nano-particles (Figs. 1, 2 and 3). The granules have a rough surface structure that is highly species-specific (Fig. 3). The secretion is present on all body parts, except regions situated close to and at joint membranes and segment borders, the distitarsi and pretarsi of legs, parts of the chelicerae and the eyes. It may also be absent on the tips of tubercles that widely cover the dorsal cuticle of the carapace and parts of the legs (except in *C. acosta*).

**Fig. 4:**
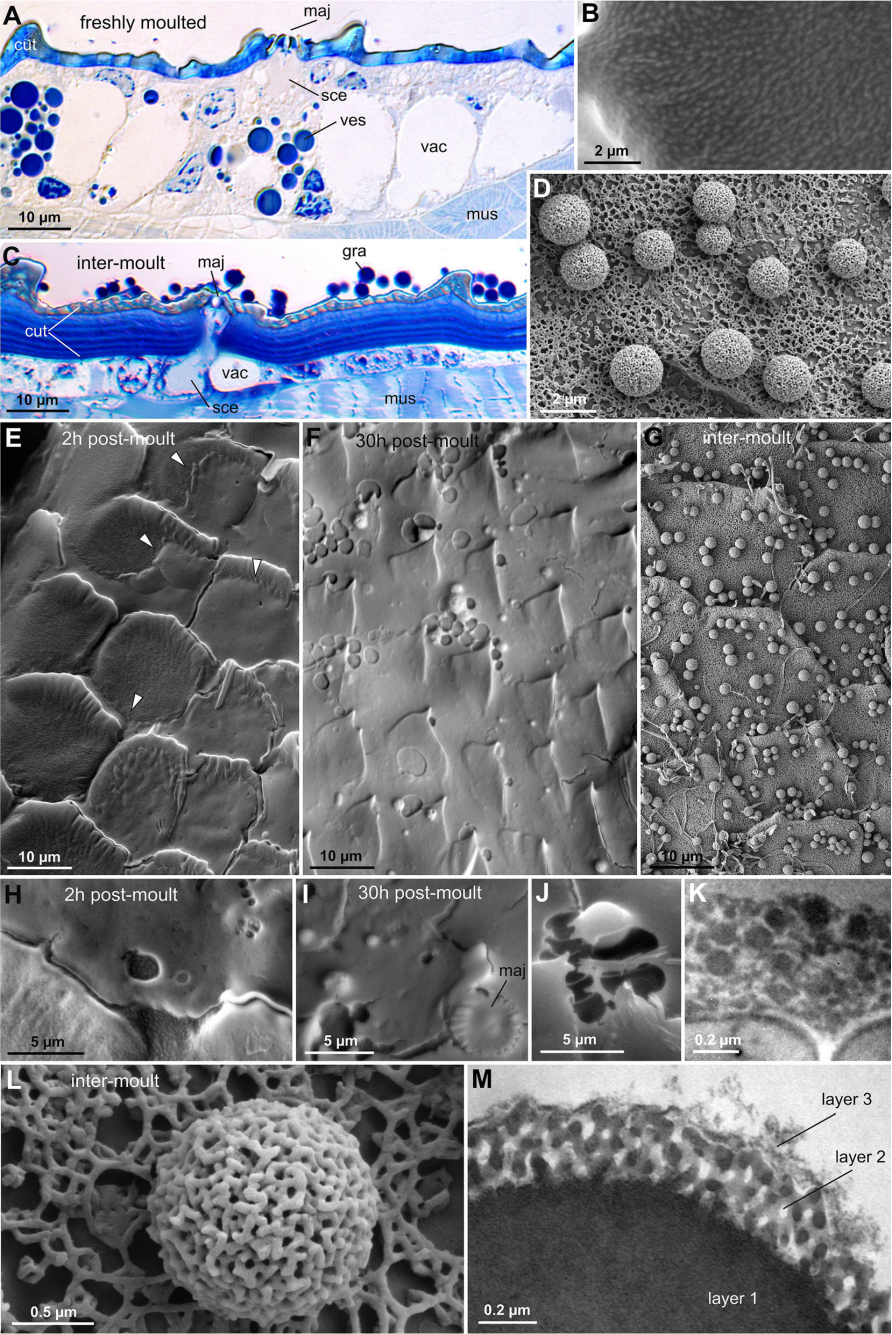
Formation of the secretion crust in *Charinus acosta*. **a**. Histological section of the integument of a freshly moulted animal (dorsal carapace). Different epidermis cells are present between the cuticle (cut) and the musculature (mus), containing either spherical strongly stained vesicles (ves) or large vacuoles (vac). The 2-lipped major gland openings (maj) are connected to a secretory cell (sce), in which the content of different vesicles is apparently mixed (see Fig. 5). **b**. An SEM micrograph of the cuticle of a freshly moulted animal shows the absence of the granular secretion coat. **c**. Histological section of the integument of an inter-moult animal (same scale as in A.). The epidermal cells are reduced in size, the vacuoles shrink, and the stained vesicles are mostly absent. The solidified secretion layer is assembled on the cuticle and consists of a thin continuous layer and globular granules (gra), both of which are highly stained like the vesicles in A. The different in the thickness of the cuticle (cut) if compared to A. is due to the difference in the animal instar. **d**. Same as in B., showing the assembled secretion crust. **e**. Appearance of the carapace cuticle at an early stage of secretion emergence (2 h after moulting). *Arrowheads* indicate the advancing secretion film. The scale-like structures are cuticular formations. **f**. Appearance of the secretion coat 30 h after moulting (leg cuticle). Globular structures are beginning to emerge. Frequent holes in the secretion film may result from vesicles of a volatile phase, presumably water. **g**. Appearance of the fully shaped secretion crust, as observed in an exuvia (carapace). **h**. Detail of the advancing secretion film 2 h after moulting. **i**–**j**. Details of the secretion film 30 h after moulting with forming granules. Note that the surface of granules is still smooth and sizes differ. **k**. TEM micrograph showing a detail of the assembled secretion film after solidification. **l**–**m**. Fine structure of a granule. Three layers are distinguishable, an isotropic inner layer, a nano-porous outer layer and 3^rd^ layer of an irregular material

TEM images of granules in *C. acosta* shows a multi-layered structure (Fig. 4m). The bulk of a granule is formed by a homogeneous material. It is covered by a porous crystal-like layer of assembled nano-particles. The outermost layer is thin and irregular and composed of a loose granular substance. All these substances are rather electron-dense. SEM observations of fractured cuticles in *C.* cf. *grayi* and *D. annulatipes* (Fig. 6) show that the inner material appears always homogeneous, and that species-specific differences in granule morphology are the result of the specific structure of the second crystal-like layer. The uppermost layer was not visible in SEM images, and may be a fluid.

### Secretion

**Fig. 5:**
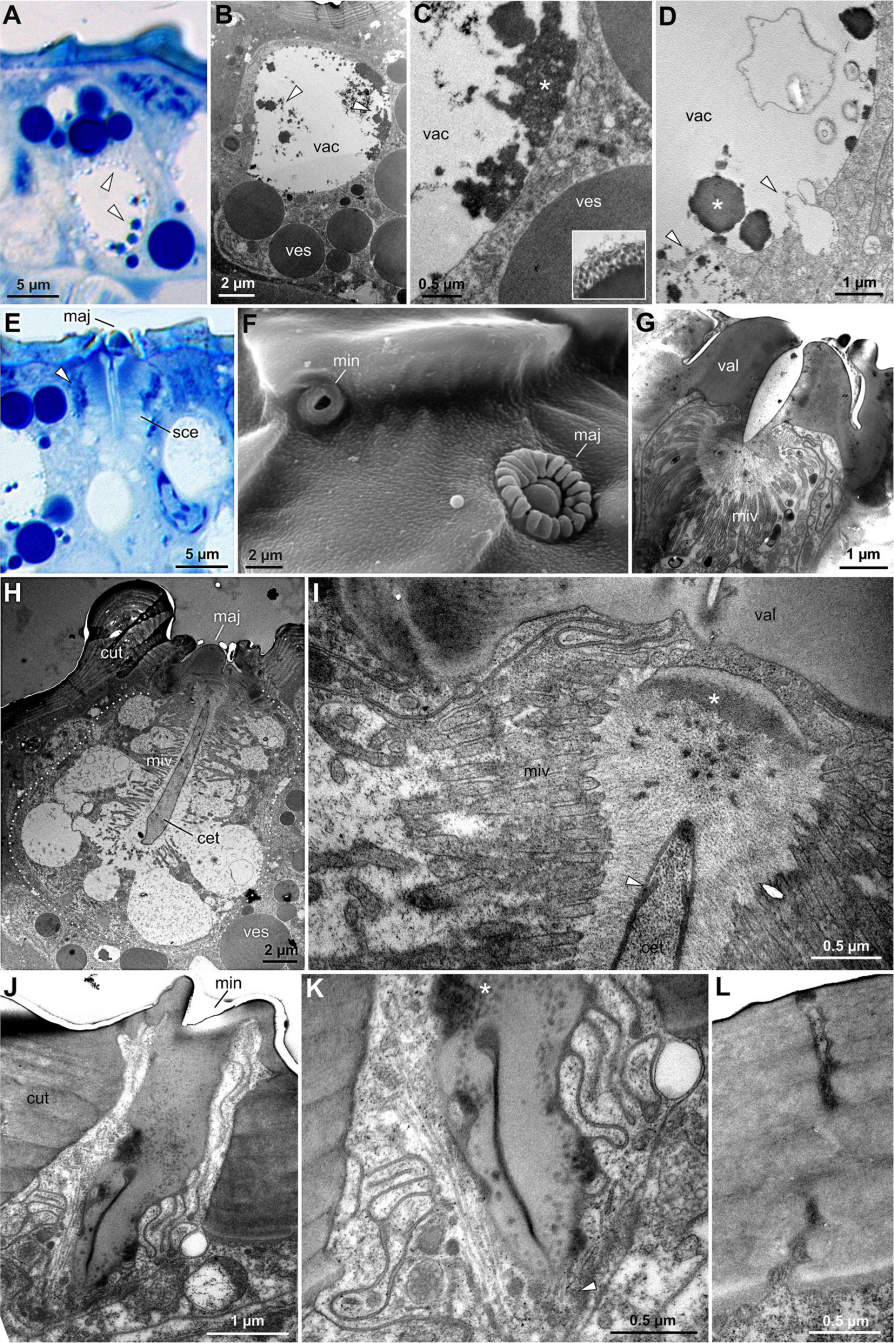
Synthesis and secretion of the coating in *Charinus acosta*. **a**-**d**. In some epidermis cells the stained vesicles (ves) merge with the vacuoles (vac) (*arrowheads*) ejecting insoluble nanoparticles (*asterisks*). **e**. The secretory cell (sce) connected with the major gland opening (maj) exhibits a vertical concentration gradient of stained secretion components. Partitions of vacuoles merge with the proximal part of the cell while small stained vesicles especially assemble at and merge with the distal part of the cell (*arrowhead*). **f**. SEM micrograph of a freshly moulted animal showing two types of gland openings: the major gland opening (maj) and the smaller pore-like minor gland opening (min). **g**. Section of the valve-like cuticle structure controlling the fluid emergence from the major gland opening, presumably by a change in the cuticular stiffness during cuticle curing after moulting. **h**. TEM image of the secretion cell connected with the major gland opening (maj) (cell borders are enhanced by *dotted line*). The cell exhibits a central tubular structure (cet) surrounded by microvilli (miv) branching of the lateral membranes of the vacuole-like structure with increasing density towards the opening. **i**. Detail of microvilli (miv) and assembling secretion (asterisk) underneath the valve-like cuticular opening (val). **j**–**k**. Secretory apparatus connected to the pore-like minor gland opening (min). The secretion fraction assembling in this cell is slightly stained and contains globular nano-particles (*asterisk*), coming from small vesicles (*arrowhead*). Many membranous folds are present in this cell. **l**. Cuticular nano-channel, containing a granular material

A high amount of secretory cells is present in the epidermis, with two main types of vesicles (Figs. 4a and 5a–b): (1) spherical vesicles whose content is highly stained, and (2) large non-stained vesicles, presumably containing water (or an aqueous solution), and therefore called ‘vacuoles’. Both vesicle types are highly reduced in number and size after the formation of the secretion coat, as is the overall thickness of the epidermis (Fig. 4c). The deposited secretion crust exhibits similar staining properties as the content of the spherical vesicles, indicating that these bear the material that forms the later coat. However, the spherical vesicles do not retain their shape and are not secreted as ready-made granules. Instead, they are fusing with the vacuoles giving off a non-soluble granular substance (Fig. 5a–d).

Two types of gland openings are evenly distributed on all parts of the cuticle that eventually bear the secretion layer (Figs. 2 and 5f). The first (major gland opening) is a slit of about 4 μm length flanked by two cuticular lips (Fig. 5g). Underneath there is a cell with a highly specific microstructure (Fig. 5e, h), which includes a large lumen that is penetrated by a central tubular structure and surrounded by microvilli. The microvilli emerge from the lateral cell membranes into the lumen and their density increases gradually towards the gland opening (Fig. 5h–i). Thus there is a concentration gradient of the solution stored in the vacuole, with increasing staining affinity towards the gland opening (Fig. 5h–i). The stainable compounds come from small spherical vesicles assembling in the distal part of the cell (Fig. 5e, h).

The second (minor gland opening) is a simple pore with a diameter of about 1 μm. The pore is connected to a cylindrical lumen that contains a slightly stained isotropic substance in which highly stained nanoparticles are dispersed (Fig. 5j–k). The surrounding cells exhibit many membrane folds within the cytoplasm, indicating high synthesis activity.

**Fig. 6:**
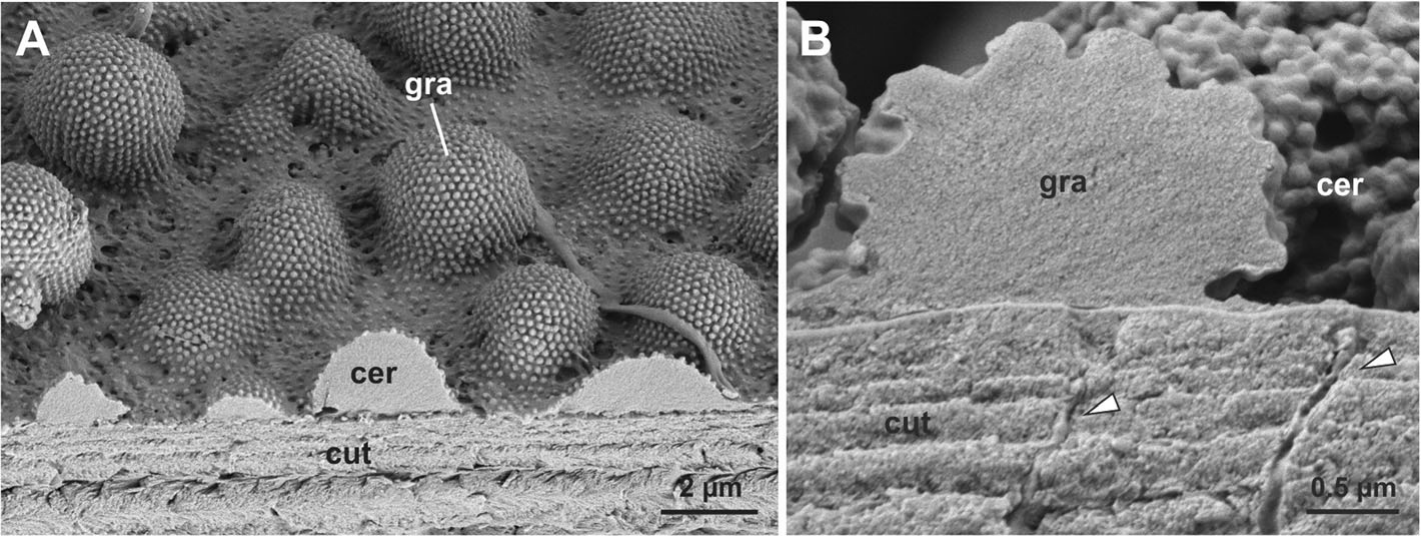
Fractures of exuviae. **a**. Fracture of an exuvia of *Charon* cf. *grayi*, showing the layered cuticle (cut) and the secretion crust (cer) with granules (gra). Note the homogeneous appearance and even fracture face of the granules indicating that the bulk material is isotropic. **b**. Same in *Damon annulatipes*. Note the cuticular nano-pores, containing some substance (*arrowheads*)

Additionally, there are pore canals (diameter of ~50 nm) (Fig. 5l), scattered throughout the cuticle. In fractures of exuviae of *D. annulatipes* these contain an isotropic material (Fig. 6b). Such pore canals are typically present in arthropod cuticle and involved in the secretion of epi-cuticular hydrocarbons [15, 23].

### Post-secretion dynamics and self-assembly

The cuticles of freshly moulted *C. acosta* and *P. longipes* show no granular structures, and their bare cuticle is rather smooth on a microscopic scale (Figs. 2 and 4d). Emerging secretion was found in some places (Fig. 4e), with a rather homogenous structure, except for some holes and dimples resulting from bubble like enclosures (Fig. 4h). In an individual of *C. acosta* killed 30 h after moulting, the secretion coat was evenly spread on the carapace and legs and exhibited globular structures apparently separating from the continuous film, and dimples left behind by the evaporation of a volatile compound (Fig. 4f, i–j). The final crust, as observed in an exuvia, showed a highly elaborate structure with rather similarly sized and evenly distributed granules covered by a crystal-like layer of assembled nanoparticles (Fig. 4g, l).

Samples from an individual of *P. longipes,* frozen 1 day after moulting followed by air drying, exhibited a thin layer of secretion on the cuticle, containing small crystals (diameter ~50 nm) and solidified droplets (diameter ~50–100 nm) (Additional file 1: Figure S3,B–D). Droplets arise from the colloidal phases of a solidifying secretion, emerging from the major gland opening, and a partly volatile secretion, emerging from the minor gland openings, that leaves dimples after evaporation (Additional file 1: Figure S3, B–D).

Once hardened, the secretion forms a stable crust that cannot be dissolved in water or ethanol. The crust can be removed in part by scratching; however, total abrasion is prevented by the tubercular structure of the cuticle.

### Wettability of the whip spider cuticle

In all species tested (completely hardened surface), water droplets dripped onto the whip spider carapace roll off (Fig. 2, Additional file 2: Video S1). Water droplets directly ejected on the carapace formed spherical shapes (Figs. 1 and 3) and could only be held in place, when placed in the dimple of the mid carapace. Movement of the animal induced the droplet to roll off, except in some individuals of *C.* cf. *grayi* and *P. longipes*. Wettability of the smooth tips of tubercles is higher; hence the droplet may contact only these parts, indicated by a visible (reflecting) air film in between and also by a partly deformation of the droplet near the contact to the solid surface. In contrast, an individual of *P. longipes* was highly wettable (total spreading of the water droplet) some hours after moulting, showing that super-hydrophobicity is caused by the secretion crust formed 1–2 days after moulting (Fig. 2, Additional file 3: Video S2).Additional file 2: Video S1: *Phrynus decoratus* showing super-hydrophobicity (playback speed: 50% of real time). (WMV 1549 kb)
 Additional file 3: Video S2: Wettability of the cuticle of *Phrynus longipes* some hours after moulting and between moultings (playback speed: 50% of real time). (WMV 2236 kb)


## Discussion

Plants and animals usually acquire super-hydrophobic surface structures through growth, self-assembly of waxes, or the deposition of nano-particles (Table 1). The mechanism, we found in whip spiders, differs in that the surface structure (and resulting super-hydrophobicity) arises from an initially homogeneous secretion coat after its extrusion onto the cuticle surface. Such an additional layer of a solidified secretion crust forming complex microstructures has previously been described as so-called *cerotegument* in mites [24] and millipedes [25], but never from arthropods as large as whip spiders. Furthermore, the secretion processes and formation of structures have never been reported to date. We found a remarkable complexity of the resulting surface: there is a micro-roughness caused by the granules arising from assembling droplets, and a nano-roughness caused by the arrangement of nano-particles at the interface between two immiscible phases, or by droplets of a volatile component leaving dimples behind. This topography leads to the enclosure of air bubbles when wetted, which further decreases the attachment of a water droplet (Cassie state) [26].

Based on our microscopic observations, we propose that there are two main secretion fractions extruded separately, with one water-based phase emerging from the major gland openings, and a another phase based on an electron-denser solvent and emerging from the pore-like minor gland openings. This is indicated both by a difference in staining behaviour and the distinct cellular structures present in both types of secretory cells. However, the exact mechanisms forming the microstructures during the long curing process remain unclear, especially since the chemical identity and physical properties of secretion fractions are not known. Water may act as a volatile medium partitioning globular fractions from the insoluble phase by specific surfactants. It is also probable that exoenzyme processes play a role, which would explain the long duration of the crust maturation.

The nano-particles that assemble on the surface of the granules and the continuous film in between form regular patterns. Such self-assembly of nanoparticles at interfaces can be driven by attractive and repulsive interactions or capillary forces during evaporation of the volatile medium, as it is applied in colloidal lithography [27]. Hierarchical globular structures with hydrophobic properties, comparable to the shape and size of granules of some whip spider species we studied (*C.* cf. *grayi* and *P. ceylonicus*) have previously been synthetically produced by multi-step colloid lithography [28]. Nonetheless, the structural diversity we observed in different species of whip spiders cannot be generated by such methods. A chemical analysis and in vivo observation of secretion assembly is necessary to shed light on the underlying mechanisms involved in the formation of such complex and highly un-wettable structures as found in whip spiders. Mimicking such coatings for technical applications may not only be of interest as self-cleaning and water-repellent surfaces, but also for a broad range of hierarchically structured, functionalized surfaces with high wear resistance.

The biological function of the super-hydrophobic coating in whip spiders is not known and rather speculation. It may be related to plastron respiration during over-flooding of the microhabitat. Many whip spider species live on the ground or in caves and have been shown to stay in a close range of their resting site [21]. Laboratory studies on the species *Phrynus marginemaculatus* have demonstrated that it can survive for more than 24 h when submerged in water, indicating the presence of a plastron [22]. Due to the Cassie state, a thin air film is formed in water and may act as a physical gill. Interestingly, the tips of tubercles often stay free of secretion and are highly wettable. Such structure may stabilize an air film, similar to the recently described *Salvinia*-effect in swimming plants [29]. In addition, the hierarchically-structured secretion layer may provide a self-cleaning effect, prevent bacterial adhesion, as well as play a role in coloration and camouflage. Due to its smoothness, the whip spider cuticle is highly reflecting and shiny, but matted by the secretion that optically appears similar to clay dust. Areas of different secretion amounts contribute to particular coloration patterns, blending the animal with its environment.

## Conclusions

These observations represent an intriguing new example of a functional biological surface and may shed new light on the biology of whip spiders. Elucidating the physico-chemical processes involved could render new ideas for the development of novel colloid-generated coating techniques, in order to achieve durable films of hierarchical microstructures on various surfaces.

## Additional files

Additional file 1: Figure S3. Formation of the secretion coat in *Phrynus longipes*. A. Bare cuticle of a *Phrynus longipes* 6 h after moulting, exhibiting tubercles (tub) and gland openings. Asterisks indicate the major gland openings, arrowheads the minor gland openings. B. Detail of minor gland opening in a specimen 24 h after moulting. Note the thin homogeneous coat on the cuticle, except in the vicinity of the pore. Note also the nano-crystals accumulating at the edge of the secretion coat (arrowheads). C. Detail of major gland opening in a specimen of *P. longipes* 24 h after moulting. Note the continuous secretion film with evenly distribution of tiny droplets of a volatile substance (arrowheads). D. Detail of the margin of a major gland opening showing formation of globular granules from a continuous phase. (JPG 719 kb)
