# Supplementary figures and images for: Whip spiders (Amblypygi) become water-repellent by a colloidal secretion that self-assembles into hierarchical microstructures
Source: Zoological Lett. 2016 Nov 28;2:23. doi: 10.1186/s40851-016-0059-y (PMC5126833; doi:10.1186/s40851-016-0059-y)

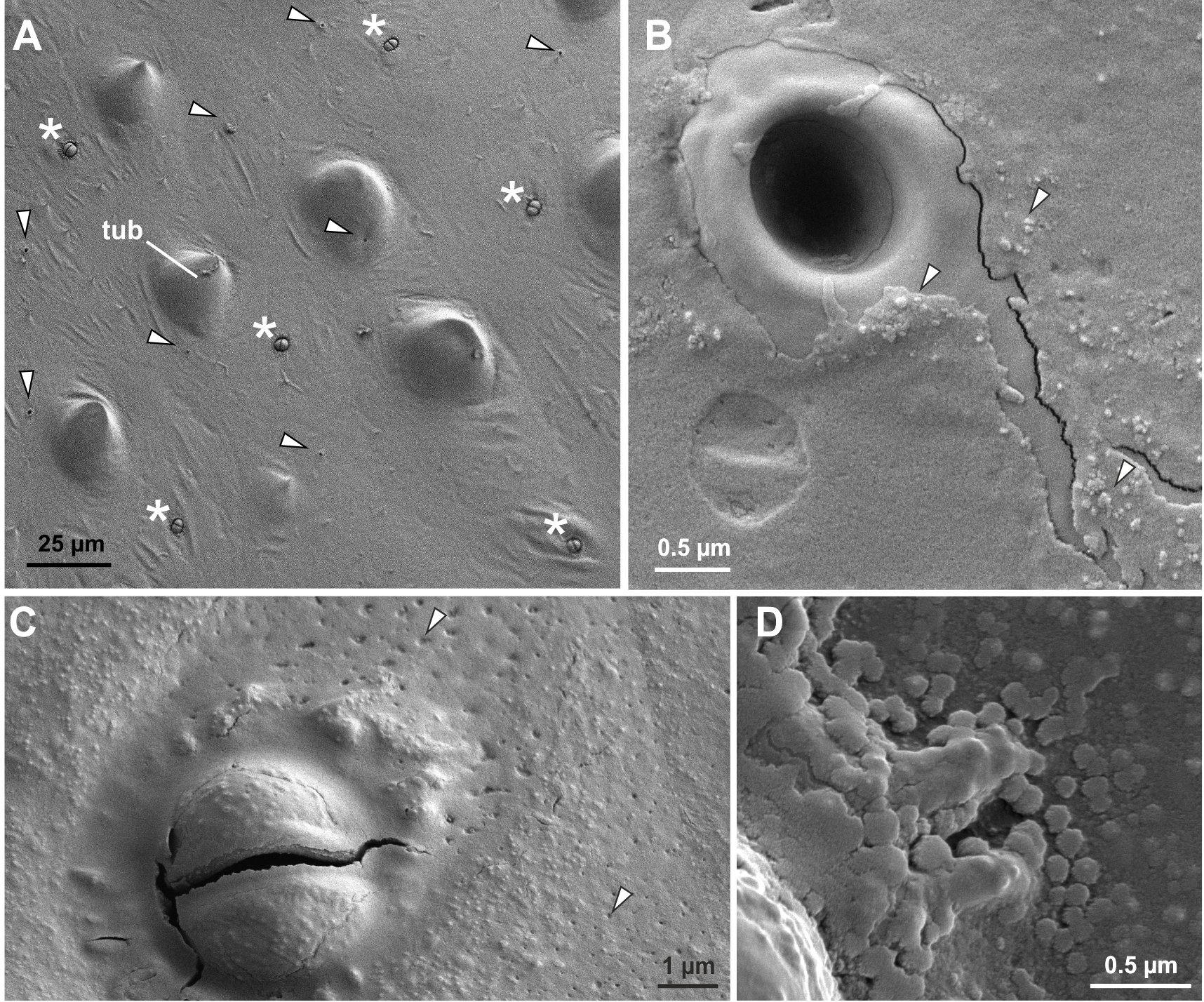

Supplement: Additional file 1: Figure S3. — Formation of the secretion coat in Phrynus longipes. A. Bare cuticle of a Phrynus longipes 6 h after moulting, exhibiting tubercles (tub) and gland openings. Asterisks indicate the major gland openings, arrowheads the minor gland openings. B. Detail of minor gland opening in a specimen 24 h after moulting. Note the thin homogeneous coat on the cuticle, except in the vicinity of the pore. Note also the nano-crystals accumulating at the edge of the secretion coat (arrowheads). C. Detail of major gland opening in a specimen of P. longipes 24 h after moulting. Note the continuous secretion film with evenly distribution of tiny droplets of a volatile substance (arrowheads). D. Detail of the margin of a major gland opening showing formation of globular granules from a continuous phase. (JPG 719 kb) [file 40851_2016_59_MOESM1_ESM.jpg]
